# Study on the Molecular Mechanism of Arbuscular Mycorrhizal Symbiosis Regulating Polysaccharide Synthesis in *Dendrobium officinale*

**DOI:** 10.3390/ijms26199298

**Published:** 2025-09-23

**Authors:** Jiadong Chen, Yiqun Zhang, Man Zhang, Ziyi Zhang, Yingying Liu, Xiaojing Duan, Zhengming Tao, Wu Jiang

**Affiliations:** 1 Zhejiang Institute of Subtropical Crops, Zhejiang Academy of Agricultural Sciences, Wenzhou 325005, China; 2 State Key Laboratory of Crop Genetics & Germplasm Enhancement and Utilization, College of Resources and Environmental Sciences, Nanjing Agricultural University, Nanjing 210095, China

**Keywords:** *Dendrobium officinale*, mycorrhizal symbiosis, polysaccharide synthesis, expressed genes, glycosyltransferase

## Abstract

Mycorrhizal symbiosis represents a ubiquitous mutualistic relationship in nature, wherein mycorrhizal fungi enhance the host plant’s ability to absorb water and nutrients from the soil. In return, the host plant supplies the fungi with essential nutrients necessary for their metabolic activities. However, research focusing on the regulatory mechanisms governing mycorrhizal symbiosis in *Dendrobium officinale* remains limited. This study systematically investigates the regulatory mechanisms of mycorrhizal symbiosis on transcriptional synthesis in *D. officinale* by establishing a mycorrhizal symbiotic system, complemented by phenotypic observation, physiological measurement, and transcriptome sequencing. The results indicate that mycorrhizal symbiosis promotes both growth and nutrient absorption in *D. officinale*, concurrently increasing polysaccharide content. Through transcriptome analysis, we identified 59 differentially expressed genes associated with polysaccharide metabolism, alongside key genes and transcription factors integral to the regulatory network. Notably, the glycosyltransferase gene *DoUGT83A1* was found to negatively regulate the mycorrhizal symbiotic system when heterologously expressed in tomato. This study provides a fundamental theoretical basis for elucidating the molecular mechanisms underlying polysaccharide synthesis in *D. officinale* and offers new insights for optimizing cultivation practices to enhance medicinal quality.

## 1. Introduction

*Dendrobium officinale* Kimura & Migo, a perennial epiphytic herb belonging to the genus *Dendrobium* in the Orchidaceae family, is a highly valued traditional Chinese medicinal herb [1]. For its ornamental value and a range of therapeutic effect, *D. officinale* is ranked as one of the ‘The first of nine Chinese fairy grasses’, and is also included in the Chinese Pharmacopoeia (2025, pp. 303–304) [2,3]. According to the “Compendium of Materia Medica,” *D. officinale* is known for its effects such as “strengthening yin and replenishing essence, supplementing internal deficiencies, enhancing intelligence and alleviating fear, and promoting longevity.” Research has identified various active components in *D. officinale*, including polysaccharides, alkaloids, and amino acids [4]. Modern pharmacological studies indicate that *D. officinale* possesses significant therapeutic effects, including enhancing human immunity, anti-aging properties, tumor inhibition, and sleep improvement [5,6]. The “Chinese Pharmacopoeia” stipulates that the mass fraction of polysaccharides in qualified *D. officinale* must not be less than 25%, highlighting the synthesis and transformation of these polysaccharides as a focal point in contemporary academic research [7]. The polysaccharides in *D. officinale* are primarily composed of glucose, mannose, xylose, rhamnose, glucuronic acid, and galacturonic acid [8]. Substantial research has been conducted on the phytochemical constituents and therapeutic mechanisms of *D. officinale*. Polysaccharides, as one of the primary bioactive components in *D. officinale*, exhibit multifaceted pharmacological effects, including immunomodulation, antitumor activity, antioxidant properties, and anti-inflammatory actions [9,10,11,12,13].

Mycorrhizae represent a symbiotic association formed between fungi and the roots of host plants, serving as a crucial mechanism for plants to adapt to environmental stressors [14,15]. Arbuscular mycorrhizal (AM) association occurs in an endosymbiotic form, characterized by the formation of highly branched hyphal structures known as arbuscules within the root cortical cells. This phenomenon is a hallmark of AM symbiosis and plays a crucial role in the interaction between the fungal partner and the host plant [16]. Mycorrhizal symbiosis aids hosts in absorbing nutrients from soil outside the rhizosphere via extrarhizosphere hyphae, thereby enhancing their uptake of essential nutrients like phosphorus, nitrogen, and potassium [17,18]. Host plants offer their own carbohydrates as the essential carbon source for the growth of mycorrhizal fungi, thereby sustaining a healthy symbiotic relationship between the two parties [19,20]. Mycorrhizal symbiosis enhances medicinal plants’ resistance to both environmental stresses and pathogen/pest infestations, while promoting the accumulation of bioactive medicinal compounds. For example, AM might play a key role in alfalfa growth and survival under harsh salt conditions [21]. Different arbuscular mycorrhizal fungal (AMF) species (particularly the mixed inoculum) improved the growth and physiological traits of *Eclipta prostrata* (L.) L. under both non-saline and saline conditions by enhancing antioxidant enzyme activity, promoting proline and total phenolic accumulation, and significantly regulating the spatiotemporal dynamics of polyphenols [22]. Previous research has demonstrated that AMF communities possess considerable potential in enhancing the yield and promoting the accumulation of bioactive components in *Salvia miltiorrhiza* Bge. [23].

This study inoculated potted *D. officinale* with mycorrhizal fungi to investigate the effects of mycorrhizal symbiosis on the growth and polysaccharide accumulation of *D. officinale*. Transcriptome sequencing was employed to analyze the transcriptional regulatory pathways associated with mycorrhizal symbiosis. A glycosyltransferase gene, *DoUGT83A1*, which is induced by mycorrhizal symbiosis, was identified. Through heterologous validation in transformed tomatoes, the role of *DoUGT83A1* in sugar metabolism and the mycorrhizal symbiotic pathways of *D. officinale* was elucidated, providing a theoretical basis for understanding the transcriptional regulatory mechanisms of mycorrhizal symbiosis in *D. officinale*.

## 2. Results

### 2.1. Influence of Mycorrhizal Fungi on Phenotypic Characteristics of D. officinale

**Figure 1:**
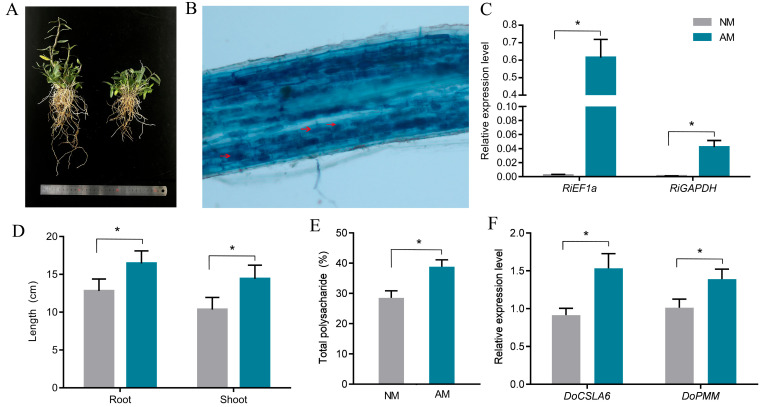
Phenotypic Analysis of Mycorrhizal Symbiosis in *D. officinale*. (**A**) Phenotypes of mycorrhizal symbiotic and non-symbiotic *D. officinale*. (**B**) Microscopic observation of stained roots of mycorrhizal symbiotic *D. officinale*, with red arrows indicating arbuscules. (**C**) Expression levels of marker genes *RiEF1a* and *RiGAPDH* in inoculated and non-inoculated roots. (**D**) Statistics on root and shoot lengths of inoculated and non-inoculated *D. officinale.* (**E**) Results of total polysaccharide content detection. (**F**) Expression levels of related to gluconeogenesis (*DoPMM, DoCSLA6)* in inoculated and non-inoculated roots. Values are means of three biological replicates with SE, * *p* < 0.05. AM: inoculation with *Rhizophagus irregularis*, NM: inoculated with an equal amount of an autoclaved inoculum. Internal reference gene: *DoActin* (GenBank: KC831582.1).

Mycorrhizal symbiosis positively influences plant growth by enhancing both vegetative development and the accumulation of metabolites. This study examined the phenotypic characteristics of mycorrhizal symbiosis in *D. officinale*, demonstrating that such symbiosis significantly promotes the growth of stems and roots. Specifically, there was a 28.51% increase in above-ground length and a 39.29% increase in below-ground length (Figure 1A,D). Observations from root staining indicated that mycorrhizal fungi invaded the roots of *D. officinale*, forming arbuscular structures (Figure 1B). Quantitative fluorescence PCR analysis revealed a significant enhancement in the expression of the fungal elongation factor gene *RiEF1a* and the glyceraldehyde-3-phosphate dehydrogenase gene (*RiGAPDH*) in mycorrhizal symbiotic roots (Figure 1C). Furthermore, polysaccharides, which are essential medicinal components of *D. officinale* and are known to enhance human immunity, were found to increase in total content due to mycorrhizal symbiosis (Figure 1E). Our findings indicate that the expression levels of *DoPMM* and *DoCSLA6*, which are essential for gluconeogenesis in *D. officinale* inoculated with mycorrhizal fungi, were significantly higher than those observed in the control group (Figure 1F). Nitrogen and phosphorus are essential elements for plant growth. Relevant studies have demonstrated that mycorrhizal symbiosis aids the host in nutrient absorption from the environment. In this study, we found that the nitrogen and phosphorus content in mycorrhizal symbiotic *D. officinale* plants was significantly higher than that in the control group (Supplementary Figure S1).

### 2.2. RNA Sequencing and Assembly of the D. officinale Transcriptome

Considering the histological differences between arbuscular mycorrhizal symbiosis (AM) and non-symbiotic (NM) roots of *D. officinale*, we collected three biological replicates of each type for the preparation of RNA-seq libraries. High-quality RNA-Seq data were obtained for each sample, yielding between 39 and 47 million unidirectional reads. In total, 273,034,882 raw reads were generated from the six libraries. After filtering out low-quality sequences, we obtained 255,989,632 clean reads, with average Q20 and Q30 values of 97.71% and 93.385%, respectively. From these data, we identified 24,023 genes, with lengths ranging from 53 to 19,125 base pairs (Table S1).

**Figure 2:**
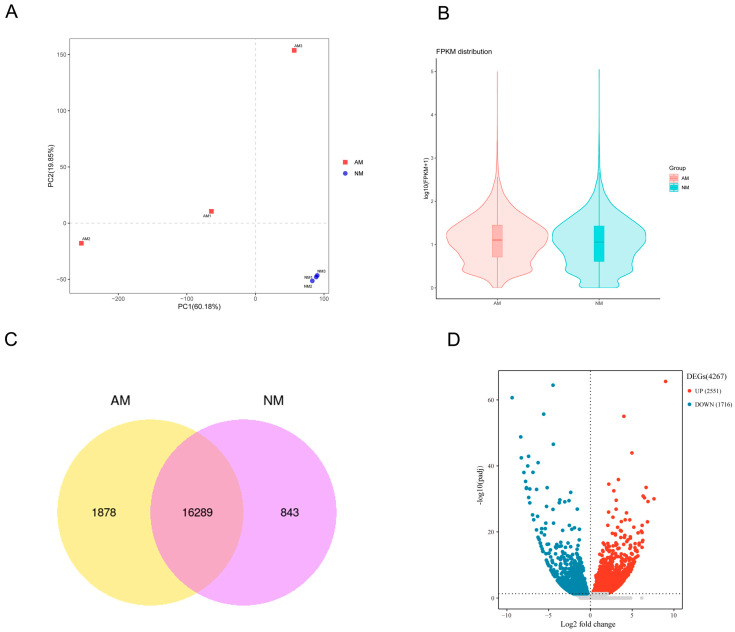
Gene expression atlas of mycorrhizal symbiosis in *D. officinale.* (**A**) Principal component analysis under two treatments is shown. (**B**) FPKM violin plots in different treatments. (**C**) Venn diagram displays the number of uniquely and overlappingly expressed genes in different treatments (AM-NM). (**D**) Volcano plot comparing the number of up- and down-regulated genes in different treatments. AM: inoculation with *Rhizophagus irregularis,* NM: inoculated with an equal amount of an inactivated microbial agent.

Principal Component Analysis (PCA) identified two distinct clusters, with the first two components accounting for 80.03% of the total variance, thereby revealing distinct mRNA populations across different types (Figure 2A). Our analysis indicated that 1978 genes in the AM group and 843 genes in the NM group exhibited highly stage-specific expression patterns, whereas the majority of genes (16,289) were commonly expressed in *D. officinale* (Figure 2C). Additionally, we evaluated gene expression profiles among biological triplicates, which demonstrated a high degree of correlation (R^2^ > 0.803) (Supplementary Figure S2).

Using a threshold of fold change (FC) > 1 and false discovery rate (FDR) < 0.05, we identified 4267 differentially expressed genes (DEGs), indicating that mycorrhizal symbiosis triggered significant alterations in the gene expression profile of *D. officinale* (Figure 2D). These results represented substantial differences in gene expression profiles across *D. officinale* mycorrhizal symbiosis. HCA using all DEGs revealed two discrete clusters corresponding to different types (Supplementary Figure S3), which constitute distinct gene sets, highlighting the specialized nature of suggesting a tight linkage of DEGs with mycorrhizal symbiosis responses.

**Figure 3:**
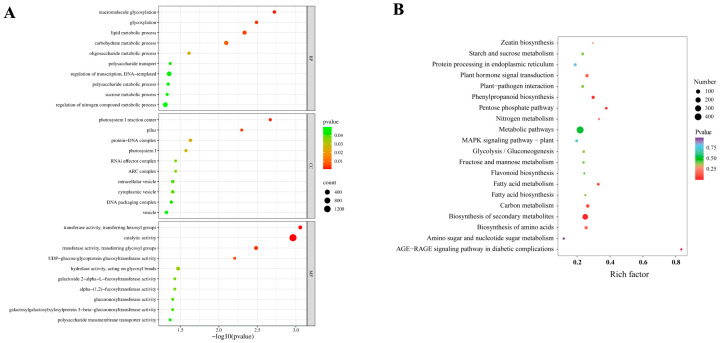
Biological Processes GO terms enrichment and The KEGG pathway analysis for upregulated and downregulated genes. (**A**) The most enriched Gene Ontology (GO) terms for all differentially expressed genes (DEGs) are categorized into three primary levels: biological process (BP), cellular component (CC), and molecular function (MF). The GO enrichment results have been transformed into negative logarithm scores to facilitate interpretation. (**B**) Enriched KEGG pathways were identified by analyzing all differentially expressed genes (DEGs) across various developmental stages using the Bioinformatics website (https://www.bioinformatics.com.cn, accessed on 15 June 2025). In the resulting figure, the columns and rows represent the enriched pathway terms and the corresponding rich factor, respectively. The size of the circles indicates the number of DEGs enriched within each specified KEGG pathway, while the color coding reflects the q-value (Corrected *p*-value) from the enrichment tests.

The enrichment analysis of differentially expressed genes (DEGs) in the Gene Ontology (GO) database revealed that, within the Biological Process (BP) category, gene alterations were predominantly concentrated in processes such as macromolecule glycosylation (GO:0043413), glycosylation (GO:0070085), lipid metabolic processes (GO:0006629), and carbohydrate metabolic processes (GO:0005975). In the Cellular Component (CC) category, gene changes were primarily focused on the photosystem I reaction center (GO:0009538), protein-DNA complexes (GO:0032993), and vesicles (GO:0031982). In the Molecular Function (MF) category, differentially expressed genes were mainly associated with transferase activity, specifically transferring hexosyl groups (GO:0016758), catalytic activity (GO:0003824), and transferase activity, specifically transferring glycosyl groups (GO:0016757) (Figure 3A). Several KEGG (Kyoto Encyclopedia of Genes and Genomes) pathways that are significantly enriched have been suggested to be linked to zeatin biosynthesis (ko00908), starch and sucrose metabolism (ko00500), plant hormone signal transduction (ko04075), and plant-pathogen interactions. (ko04626) (Figure 3B). In the study of plant hormone signal transduction, a total of 57 differentially expressed genes were identified (Supplementary Figure S4). Additionally, 35 differentially expressed genes were discovered in the context of plant-pathogen interactions (Supplementary Figure S5). Collectively, these results elucidate the regulatory effects of mycorrhizal symbiosis on the transcriptome of *D. officinale*, providing preliminary insights into the underlying biological processes.

### 2.3. Key Genes and Metabolic Pathways Involved in Polysaccharide Biosynthesis in D. officinale

**Figure 4:**
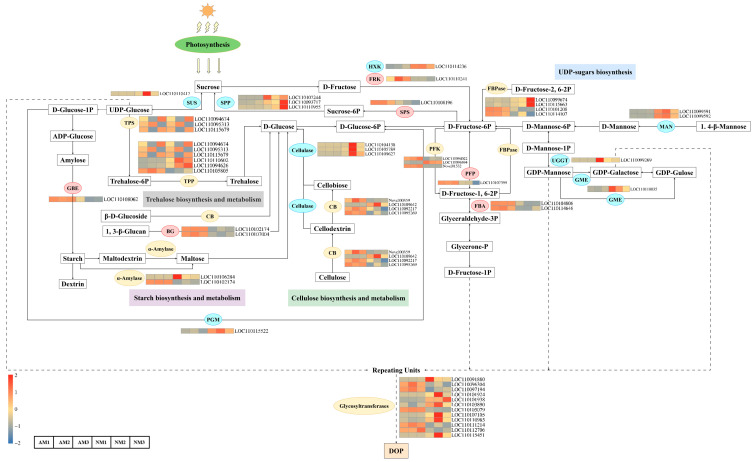
The expression patterns of genes in the polysaccharide biosynthesis pathway of *D. officinale*. The grids in different colors indicate the relative expression levels.

The biosynthesis of polysaccharides in *Dendrobium officinale* is closely linked to carbohydrate metabolic pathways, playing crucial roles in physiological processes such as photosynthesis. In the sugar metabolism-related pathways, we identified a total of 59 DEGs, which are primarily involved in three biological processes: starch biosynthesis and metabolism, cellulose biosynthesis and metabolism, and UDP-sugar synthesis. These processes may significantly influence the biosynthesis of polysaccharides in *D. officinale* (Figure 4). Our results indicate that the starch and sucrose metabolism pathway (ko00500) contains the highest number of differentially expressed genes (DEGs) (Table S2).

Compared to the non-inoculated control, the inoculation treatment significantly enhanced the activities of glucan endo-1,3-beta-D-glucosidase (BG, EC 3.2.1.39), fructose-bisphosphate aldolase (FBA, EC 4.1.2.13), sucrose phosphate synthase (SPS, EC 2.4.1.14), 1,4-alpha-glucan branching enzyme (GBE, EC 2.4.1.18), fructokinase (FRK, EC 2.7.1.4), and pyrophosphate-fructose 6-phosphate 1-phosphotransferase (PFP, EC 2.7.1.90). These enzyme activities were positively correlated with the increase in polysaccharides. Conversely, the inoculation treatment reduced the activities of sucrose phosphate phosphatase (SPP, EC 3.1.3.24), mannosidase (MAN, EC 3.2.1.78), sucrose synthase (SuSy, EC 2.4.1.13), hexokinase (HXK, EC 2.7.1.1), UDP-glucose glycosyltransferase (UGGT, EC 2.7.7.13), GDP-D-mannose 3′, 5′-epimerase (GME, EC 5.1.3.18), and phosphoglucomutase (PGM, EC 5.4.2.2), with the activities of these enzymes decreasing as polysaccharide levels increased. Additionally, the treatment altered the activities of TPP (EC 3.1.3.12), FBPase (EC 3.1.3.11, EC 3.1.3.46), CB (EC 3.2.1.21, EC 3.2.1.4), TPS (EC 2.4.1.15), PFK (EC 2.7.1.11), α-amylase (EC 3.2.1.1) (Table S3). These changes demonstrate that the inoculation treatment significantly influenced the polysaccharide biosynthesis process in *D. officinale* through the coordinated regulation of multiple enzyme activities.

A total of twelve differentially expressed glycosyltransferase genes were identified in response to arbuscular mycorrhizal fungal (AMF) colonization. Among these, seven genes, including *LOC110107015* and *LOC110115451*, were downregulated following fungal inoculation, while five genes, such as *LOC110112706* and *LOC110105079*, exhibited upregulated expression (Table S4). Quantitative real-time PCR analysis demonstrated that the relative expression level of *LOC110105079* was significantly higher than that of the other four glycosyltransferase genes (Supplementary Figure S6). Consequently, *LOC110105079* was selected as the target gene for further functional characterization. The differential expression patterns of these genes indicate their potential roles in the biosynthesis of polysaccharides in *D. officinale* and may contribute to the increased polysaccharide content observed following mycorrhizal symbiosis.

### 2.4. Identification of Transcription Factors Responsive to D. officinale Mycorrhizal Symbiosis

Transcriptome analysis of *D. officinale* before and after inoculation treatment identified a total of 2010 annotated transcription factor sequences belonging to 56 transcription factor families, (Table 1). Among these families, the bHLH family (218), MYB-related family (121), NAC family (118), WRKY family (98), ERF family (94), and B3 family (94) were identified as the most abundant transcription factors. Among the 12 glycosyltransferase genes identified previously, 9 are potentially capable of interacting with various transcription factor families. Specifically, LOC110105079, LOC110091880, LOC110096304, LOC110101924, LOC110101938, LOC110110985, and LOC110111214 may interact with the Heat Shock Factor (HSF) family, LOC110103890 may interact with the Ethylene Response Factor (ERF) family, and LOC110112706 may interact with the SQUAMOSA Promoter Binding Protein (SBP) family (Table S4).

### 2.5. Overexpression of DoUGT83A1 Inhibits Tomato Growth and Reduces Polysaccharide Synthesis in Mycorrhizal Symbiosis

**Figure 5:**
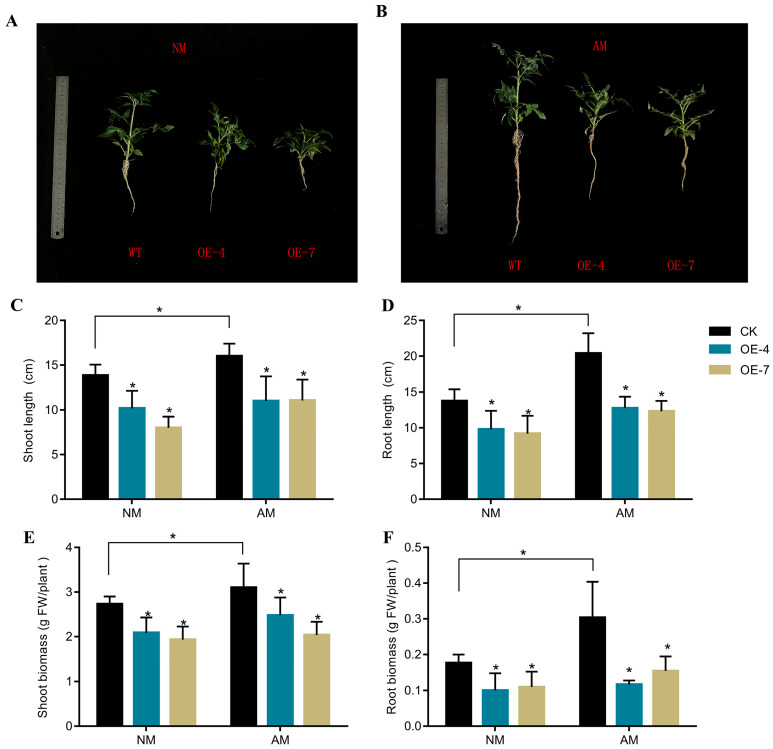
Phenotypic Analysis of *DoUGT83A1* Overexpression in Tomato. (**A**,**B**) Phenotypic images depicting the overexpression of *DoUGT83A1* in tomato plants are presented. (**A**) illustrates the phenotype without the inoculation of mycorrhizal fungi, (**B**) shows the phenotype following inoculation. (**C**,**D**) Statistical analyses of shoot (**C**) and root (**D**) lengths in the overexpressing tomato phenotype. (**E**,**F**) Statistical analyses of shoot (**E**) and root (**F**) biomass in the overexpressing tomato phenotype. Values are means of three biological replicates with SE. * *p* < 0.05. AM: inoculation with *Rhizophagus irregularis,* NM: inoculated with an equal amount of an inactivated microbial agent.

Through NCBI alignment, Gene ID *LOC110105079* was identified as *DoUGT83A1*. To investigate the role of *DoUGT83A1* in the synthesis of polysaccharides in *D. officinale* and its regulatory pathway in mycorrhizal symbiosis, transgenic tomato plants were generated to constitutively overexpress *DoUGT83A1* under the control of the cauliflower mosaic virus 35S promoter. A total of 10 overexpression lines were established, and as anticipated, these transgenic plants exhibited high levels of *DoUGT83A1* expression (Supplementary Figure S7). Consequently, two independent transgenic lines, *UGT83A1*-OE4 and *UGT83A1*-OE7, were selected for further investigation. Compared to wild-type plants, the overexpression of *DoUGT83A1* significantly inhibited tomato growth (Figure 5A,B), with the length and weight of the inoculated mycorrhizal shoots and roots being notably lower than those of the control plants (Figure 5C–F).

**Figure 6:**
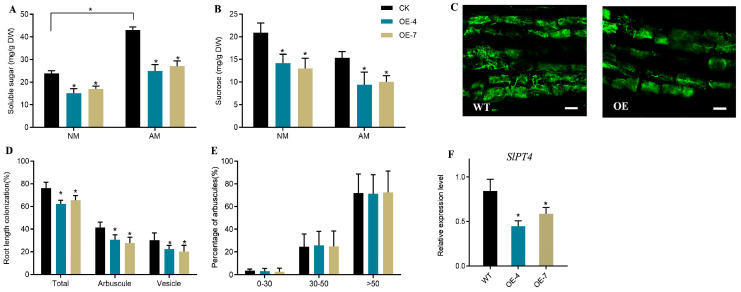
Arbuscular mycorrhizal fungal colonization analysis of the *DoUGT83A1*-overexpressing plants. WT and two *DoUGT83A1*-overexpressing lines, OE4 and OE7, were inoculated with *Rhizophagus irregularis* for 5 weeks, and then (**A**) soluble sugar content, (**B**) sucrose content, (**C**) arbuscule morphology, (**D**) the fungal colonization level, (**E**) arbuscule populations, (**F**) the transcripts of the arbuscular mycorrhizal marker gene *SlPT4*. Values are means of three biological replicates with SE. Asterisks indicate significant differences. * *p* < 0.05. (Scale bars: 50 μm). AM: inoculation with *Rhizophagus irregularis,* NM: inoculated with an equal amount of an inactivated microbial agent. Internal reference gene: SlActin (LOC105127547).

After five weeks of *R. irregularis* inoculation, the soluble sugar and sucrose contents in wild-type (WT) and *UGT83A1*-OE lines were assessed. Our results indicated that, irrespective of mycorrhizal fungal inoculation, the soluble sugar and sucrose levels in *UGT83A1*-OE plants were significantly lower than those observed in the control group (Figure 6A,B). Additionally, the arbuscular mycorrhizal (AM) colonization levels were evaluated. Although the *UGT83A1*-OE lines were capable of being colonized by AM fungi, the arbuscular colonization rate in these plants was significantly lower compared to WT plants (Figure 6D). However, no significant differences were observed in the morphology and fully developed arbuscular mycorrhizal structures between the *UGT83A1*-OE lines and WT plants (Figure 6C,E). Furthermore, the transcriptional levels of the AM-specific SlPT4 in the mycorrhizae of *UGT83A1*-OE lines were significantly reduced when compared to WT plants (Figure 6F).

## 3. Discussion

### 3.1. Establishment of Mycorrhizal Symbiosis System in D. officinale Reveals Molecular Insights into Plant-Fungal Interactions

Mycorrhizal symbiosis is widely observed in nature. Mycorrhizal fungi facilitate the absorption of water and nutrients from the soil by their host plants, in return, the host plants supply the fungi with essential sugars and fatty acids necessary for their vital activities [24,25,26]. *D. officinale* is particularly dependent on this fungal symbiosis. The application of mycorrhizal fungi is critically important for enhancing both the yield and quality of *D. officinale*; however, the underlying molecular mechanisms governing their interaction remain poorly understood.

It is well established that the development of AM symbiosis relies on continuous signaling and nutrient exchange between the host root system and AM fungi. Recent studies have highlighted the crucial roles of plant hormones, such as strigolactones, auxins, and gibberellins, in regulating AM symbiosis [27,28,29]. Additionally, flavonoids serve as significant regulators in AM symbiosis [30]. This study reveals that the mycorrhizal symbiosis of *D. officinale* induces alterations in the genes associated with the host’s hormone transcription pathways, including those involved in the synthesis of auxins (25), cytokinins (4), gibberellins (3), abscisic acid (7), ethylene (3), jasmonic acid (5), and salicylic acid (7) (Supplementary Figure S4). This suggests that *D. officinale* may respond to the symbiosis with mycorrhizal fungi through changes in plant hormones.

### 3.2. Transcriptomic Analysis Reveals AMF-Induced Modulation of Sugar Metabolism Pathways in D. officinale

Carbohydrates are products of plant photosynthesis and serve as essential components for energy supply and signaling molecules during the mycorrhizal symbiosis process. According to the 2020 edition of the ‘Chinese Pharmacopoeia,’ the polysaccharide content in *D. officinale* must not be less than 25%, while the mannose content should range from 13.0% to 38.0% [13]. The synthesis of polysaccharides in *D. officinale* is a delicate and complex process that involves a series of substance and signal interactions, along with the regulation of gene expression [31]. Previous studies have shown that polysaccharides are composed of monosaccharides, including glucose, mannose, and galactose. Notably, DoPMM and DoCSLA6 have been identified as key enzymatic genes involved in the fructose and mannose metabolic pathways [32,33]. In this study, we observed that the expression levels of *DoPMM* and *DoCSLA6* in mycorrhizal symbiotic *Dendrobium officinale* were significantly higher than those in the control group.

Current research on the functional genes of *D. officinale* identifies several genes involved in polysaccharide metabolism and transport, including UDP-Glucose Pyrophosphorylase (UGPase), Cellulose Synthase-Like A6, Sucrose Transporter, GDP-Mannose Pyrophosphorylase, DoSWEET Protein, Alkaline/Neutral Invertase Hexose Transporter, and Soluble Acid Invertase [34].

This study conducted a transcriptome analysis of *D. officinale* before and after inoculation, revealing a total of 33 differentially expressed genes that were enriched in the starch and sucrose metabolic pathways. Additionally, 17 other tertiary pathways related to polysaccharide synthesis were identified, including glycolysis/gluconeogenesis, the pentose phosphate pathway, pyruvate metabolism, and fructose and mannose metabolism. Notably, the genes exhibiting significantly upregulated expression included sucrose phosphate synthase (SPS), fructokinase (FRK), endo-1,3-β-D-glucanase (BG), and β-glucosidase (CB) (Table S2).

### 3.3. Transcriptional Regulation of Symbiotic Responses and Stress Adaptation

Transcription factors are critical regulators of gene expression, directly influencing various physiological and biochemical responses in plants. This study found that the expression levels of transcription factors such as WRKY, NAC, bZIP, and MYB-related proteins changed following inoculation treatment, indicating that the host plant responds to the invasion of exogenous microorganisms by regulating the expression of numerous genes. Members of the GRAS transcription factor family have been identified as genes induced by arbuscular mycorrhizal (AM) fungi, with tomato *SlGRAS18* interacting with *SlDELLA*, a central regulator of AM formation, thereby participating in the establishment of AM symbiosis [35]. TMLs are involved in regulating AM symbiosis, with MtTML1 and MtTML2 in alfalfa coordinating the regulation of AM colonization, as evidenced by a slight increase in AM colonization observed in mutants lacking both TMLs [36]. In this study, the analysis of transcription factors (TFs) revealed that the Heat Shock Transcription Factor (HSF) family is most closely associated with the expression of *DoUGT83A1*. Heat shock proteins (HSPs) are a class of stress-related proteins that are either newly synthesized or upregulated in organisms when exposed to adverse environmental conditions. They play a crucial role in the short-term adaptation of plants to stress. Li found that the expression of the *HSP70* gene in *Dendrobium officinale* was significantly upregulated under 4 °C conditions [37]. It is well established that the polysaccharide content in *Dendrobium officinale* significantly increases under low-temperature conditions [38].

### 3.4. DoUGT83A1 Functions as a Negative Regulator in Modulating AM Symbiosis

**Figure 7:**
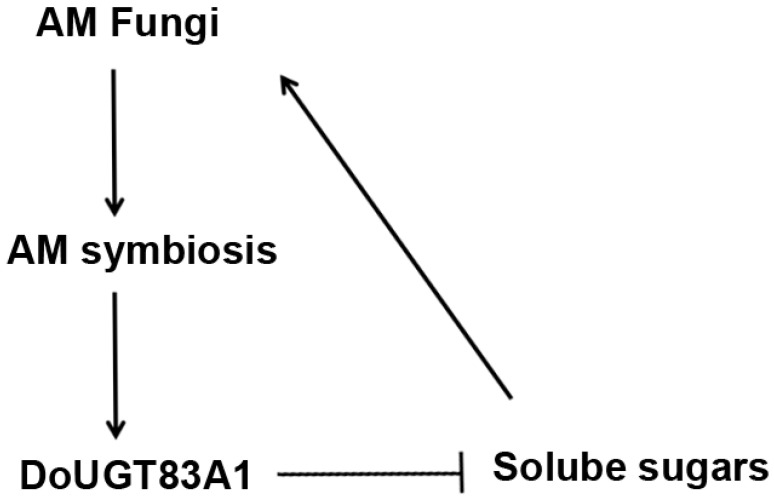
Schematic model for *DoUGT83A1*-mediated regulation of AM symbiosis. Positive and negative regulatory effects are indicated by arrows and flat-ended lines, respectively.

Glycosyltransferases represent a crucial gene family involved in the polysaccharide synthesis pathway in plants, typically functioning downstream within this pathway. Analyzing transcriptomic data from adult and seedling *Dendrobium officinale*, Zhang et al. [39] revealed that glycosyltransferase genes are critically linked to polysaccharide synthesis in this species. Additionally, Zhang et al. [40] found that the glycosyltransferase gene family is associated with the modification processes following polysaccharide synthesis in *Dendrobium officinale*. In our study, we observed significantly lower levels of soluble sugars and sucrose in the *DoUGT83A1*-OE strain compared to the control (Figure 6A,B). These sugars are essential substances that plants provide to maintain the mycorrhizal symbiotic state. Furthermore, we found that the colonization rate of mycorrhizae in the *DoUGT83A1*-OE strain was significantly lower than that of the control (Figure 6D), although the morphology and size of the arbuscules were altered (Figure 6C,E). This alteration may stem from the host plant’s efforts to dynamically regulate fungal proliferation levels, preventing excessive carbon consumption and thereby sustaining the mutualistic relationship between the two symbionts. Recently, an increasing number of components involved in arbuscular mycorrhiza self-regulation (AOM) have been identified [41,42]. We speculate that plants may develop the ability to manipulate various self-regulatory pathways to coordinate fungal colonization levels and symbiotic nutrient exchange. Based on these findings, we propose a working model (Figure 7).

## 4. Materials and Methods

### 4.1. Experimental Materials

The plant material utilized in this experiment comprised one-year-old acclimatized seedlings of the newly developed *D. officinale* cultivar ‘Tiefeng No.1’, bred by our research group. Tiefeng No.1 is a purple-stemmed D. officinale variety which contains abundant anthocyanins and polysaccharides in its stems and leaves and has excellent cold tolerance and robust disease resistance. The arbuscular mycorrhizal fungus *Rhizophagus irregularis*, employed in the study, was propagated in our laboratory to produce an inoculant containing mycelium, sporocarps, spores, yellow sand, quartz sand, and dried plant root segments. The containers used for potting were plastic flower pots with the following specifications: height 14.0 cm, upper diameter 23.0 cm, and lower diameter 11.8 cm, which were disinfected by wiping with 75% ethanol. The substrate applied was cedar sawdust, which underwent dry-heat sterilization at 180 °C for 24 h in a high-pressure steam sterilizer prior to the experiment.

### 4.2. Experimental Design

The pot experiment was conducted using a completely randomized design, incorporating two treatments: inoculation with *Rhizophagus irregularis* (AM) and a control group inoculated with an equal amount of autoclaved inoculum (NM). Each treatment was clearly labeled and replicated in five pots, with five plants per pot. The plants were cultivated in the laboratory for 90 days under alternating conditions of 28 °C for 14 h of light and 22 °C for 10 h of darkness. Watering was performed quantitatively as needed, and a 25 μM Hoagland nutrient solution was applied monthly. Watering continued until the potting substrate was thoroughly moist, following the method described by Liu et al. [43]. After three months of symbiotic cultivation, 10–15 *D. officinale* seedlings from both AM and NM treatments were randomly selected. The root substrate was rinsed away, and the roots were dried using absorbent paper. Plant height (cm) and whole plant fresh weight (g) were measured, and the average values were calculated.

### 4.3. Extraction of Total RNA and cDNA Synthesis from D. officinale 

Ninety-day-old whole seedlings of *D. officinale* were collected, with three biological replicates each for arbuscular mycorrhizal (AM) and non-mycorrhizal (NM) groups (denoted as AM1/AM2/AM3 and NM1/NM2/NM3, respectively), totaling six samples. Total RNA was isolated using a modified CTAB method [37].

RNA concentration was quantified using a Nanodrop spectrophotometer (Thermo Scientific, Waltham, MA, USA), and single-stranded cDNA was synthesized by reverse transcription following the manufacturer’s protocol of the All-in-One First-Strand Synthesis MasterMix kit (Vazyme, Nanjing, China). The samples were flash-frozen in liquid nitrogen for 30 min and stored at −80 °C for subsequent transcriptomic analysis. Relative expression level of each gene was normalized to the Actin gene (GenBank: KC831582.1) [32].

### 4.4. Transcriptome Sequencing

The transcriptome sequencing of six samples (including AM-inoculated and non-mycorrhizal (NM) treatments of *D. officinale*) was performed by Shanghai Majorbio Bio-pharm Technology Co., Ltd. The raw reads were aligned to the *D. officinale* reference genome (ASM160598v2) using the STAR aligner, and the mapped reads were visualized using the Integrative Genomics Viewer (IGV). Transcript assembly was conducted using Cufflinks (V2.2.1), followed by comparison with the reference annotation via Cuffcompare (V2.0.1) to statistically evaluate the alignment results. The mapping rate was assessed to determine whether the sequencing data met the requirements for subsequent analyses.

### 4.5. Screening and Functional Annotation of Differentially Expressed Genes (DEGs)

Gene expression levels were quantified using the FPKM method. Differentially expressed genes (DEGs) were identified with DESeq2 software (V1.36.0) using the threshold criteria of padj < 0.05 and |log2FC| > 1. Functional annotation and enrichment analysis of DEGs were performed through GO and KEGG databases. Additionally, sequencing data were subjected to alignment analysis against the GO and KEGG databases.

### 4.6. Construction of Binary Vectors and Generation of Transgenic Plants

To construct the *DoUGT83A1* overexpression vector, the open reading frame of *DoUGT83A1* was cloned into the binary vector pCAMBIA1305 using the ClonExpress II One Step Cloning Kit (Vazyme, Nanjing, China). The resulting construct, in which *DoUGT83A1* is regulated by the constitutive Cauliflower mosaic virus 35S promoter, was introduced into the *Agrobacterium tumefaciens* EHA105 strain and subsequently used for tomato transformation, as previously described [44].

### 4.7. RNA Extraction and Real-Time RT-PCR Analysis of Transgenic Tomato Lines

RNA extraction and real-time RT-PCR were conducted as described by Chen et al. [45]. Briefly, total RNA was extracted from plant tissues with the TRIzol reagent (Vazyme, Nanjing, China), and residual DNA was eliminated by treatment with DNaseI. Reverse transcription and Real-time RT-PCR were performed using the HiScript II One Step qRT-PCR SYBR Green Kit (Vazyme, Nanjing, China) with gene-specific primers listed in Table S5. Relative expression level of each gene was normalized to the Actin gene (U60481) [46].

### 4.8. Mycorrhizal Quantification and Analysis of Arbuscule Populations

For mycorrhizal quantification, root samples were stained with Trypan blue, and the level of fungal colonization was quantified using the gridline intersect method [47]. The measurement of arbuscule sizes within the arbuscule populations was conducted following the procedures outlined by Liu et al. [43]. To visualize the fungal structures, roots were stained in a 0.2 mg/mL solution of WGA AlexaFluor 488.

### 4.9. Statistical Analysis

All the data were analyzed for variance with the IBM SPSS Statistics 25.0 software, followed by Turkey’s test (*p* < 0.05), to test significant differences between different plant genotypes and treatments. The statistical significance (*p* value) was indicated in the figure legends, * *p* < 0.05, ** *p* < 0.01 and *** *p* < 0.001.

## 5. Conclusions

This study systematically investigates the regulatory mechanism of mycorrhizal symbiosis on the transcript synthesis of *D. officinale* by establishing a mycorrhizal symbiotic system, combined with phenotypic observation, physiological measurement, and transcriptome sequencing. The results indicate that mycorrhizal symbiosis promotes the growth and nutrient absorption of *D. officinale*, while also increasing polysaccharide content. Through transcriptome analysis, we identified 59 differentially expressed genes related to polysaccharide metabolism, along with key genes and transcription factors involved in the regulatory network. A glycosyltransferase gene, *DoUGT83A1*, which is up-regulated by mycorrhizal symbiosis, was screened, and its function was heterologously verified in transgenic tomatoes. Overexpression of *DoUGT83A1* resulted in a reduction in soluble sugars and sucrose content in tomatoes, as well as a decreased colonization rate of mycorrhizal symbiosis. This suggests that *DoUGT83A1* negatively regulates the mycorrhizal symbiotic system of *D. officinale*. Specifically, mycorrhizal fungi and *D. officinale* roots significantly enhance plant growth and nitrogen/phosphorus absorption, thereby providing ample nutrients and energy for polysaccharide biosynthesis. Mycorrhizal colonization alters the expression levels of genes related to sugar metabolism, particularly upregulating key enzymes involved in the synthesis and conversion of sucrose and fructose, thus supplying abundant substrates for polysaccharide production. Notably, the glycosyltransferase gene *DoUGT83A1* plays a negative regulatory role in this process. This study provides a fundamental theoretical basis for further elucidating the molecular mechanisms of polysaccharide synthesis in *D. officinale* and offers new insights for optimizing cultivation practices and enhancing medicinal quality.

## Figures and Tables

**Table 1:** Classification and Quantity of Transcription Factors.

Serial Number	Family Name	Number of Items	Serial Number	Family Name	Number of Items
1	bHLH	218	29	GATA	21
2	MYB_related	121	30	GeBP	20
3	NAC	118	31	Dof	15
4	WRKY	98	32	NF-YC	15
5	B3	94	33	SBP	13
6	ERF	94	34	AP2	12
7	bZIP	88	35	NF-YA	12
8	C2H2	86	36	NF-X1	11
9	MYB	85	37	BES1	10
10	FAR1	84	38	CO-like	10
11	G2-like	68	39	CPP	9
12	C3H	60	40	ARR-B	8
13	HD-ZIP	54	41	BBR-BPC	8
14	ARF	52	42	SRS	8
15	M-type	49	43	EIL	7
16	HSF	48	44	ZF-HD	7
17	GRAS	39	45	GRF	6
18	TCP	38	46	STAT	6
19	Nin-like	36	47	WOX	6
20	MIKC	35	48	DBB	5
21	S1Fa-like	35	49	HB-PHD	4
22	TALE	33	50	CAMTA	3
23	E2F/DP	28	51	LSD	3
24	Trihelix	28	52	HRT-like	2
25	HB-other	25	53	RAV	2
26	NF-YB	24	54	LFY	1
27	LBD	23	55	VOZ	1
28	YABBY	23	56	Whirly	1

## Data Availability

Data are contained within the article and Supplementary Materials.

## Supplementary Materials

The following supporting information can be downloaded at: https://www.mdpi.com/article/10.3390/ijms26199298/s1.
