# Supplementary material for: Study on the Molecular Mechanism of Arbuscular Mycorrhizal Symbiosis Regulating Polysaccharide Synthesis in Dendrobium officinale
Source: Int J Mol Sci. 2025 Sep 23;26(19):9298. doi: 10.3390/ijms26199298 (PMC12524374; doi:10.3390/ijms26199298)
Supplement: Supplementary file 1 [file ijms-26-09298-s001.zip › ijms-3849261-supplementary.pdf]

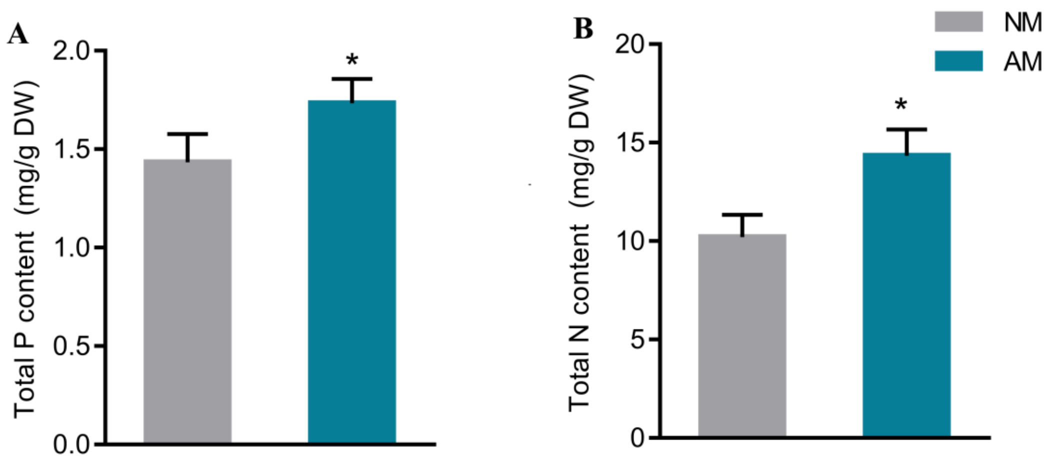

Supplementary Figure S1. Total nitrogen (**A**) and total phosphorus (**B**) content in *Dendrobium officinale*.

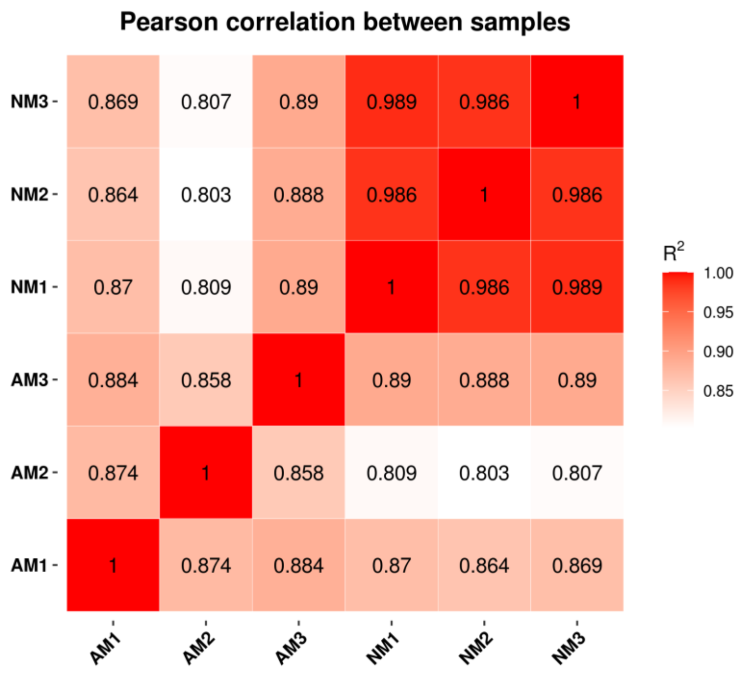

Supplementary Figure S2. Pearson's correlation matrix between biological replicates using corR package. Three independent biological replicates for between arbuscular mycorrhizal symbiosis(AM) and non-symbionts of Dendrobium officinale roots(NM) were used to construct RNA-seq libraries. Based on all gene expression data, Pearson correlation coefficients are highly correlated (R2＞0.803) between biological replicates, indicating that all collected samples for each stage were well processed.

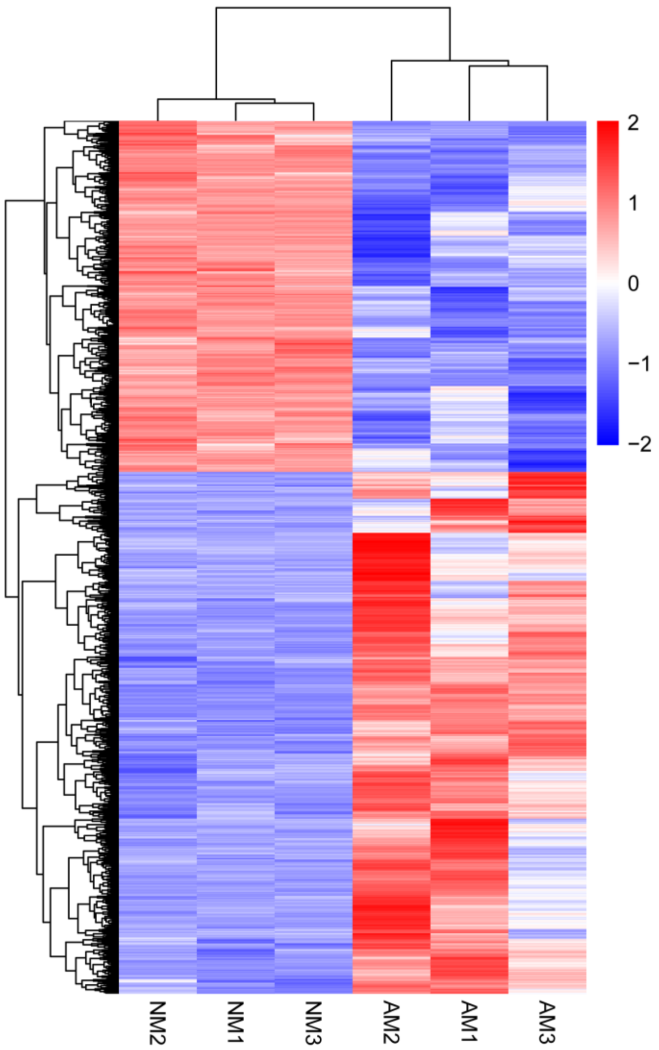

Supplementary Figure S3. Hierarchical clustering of all DEGs across Dendrobium officinale mycorrhizal symbiosis. Each row of the heat map represents an individual gene. The gene expression levels are standardized into Z-score and colored in red and blue for high and low expression.

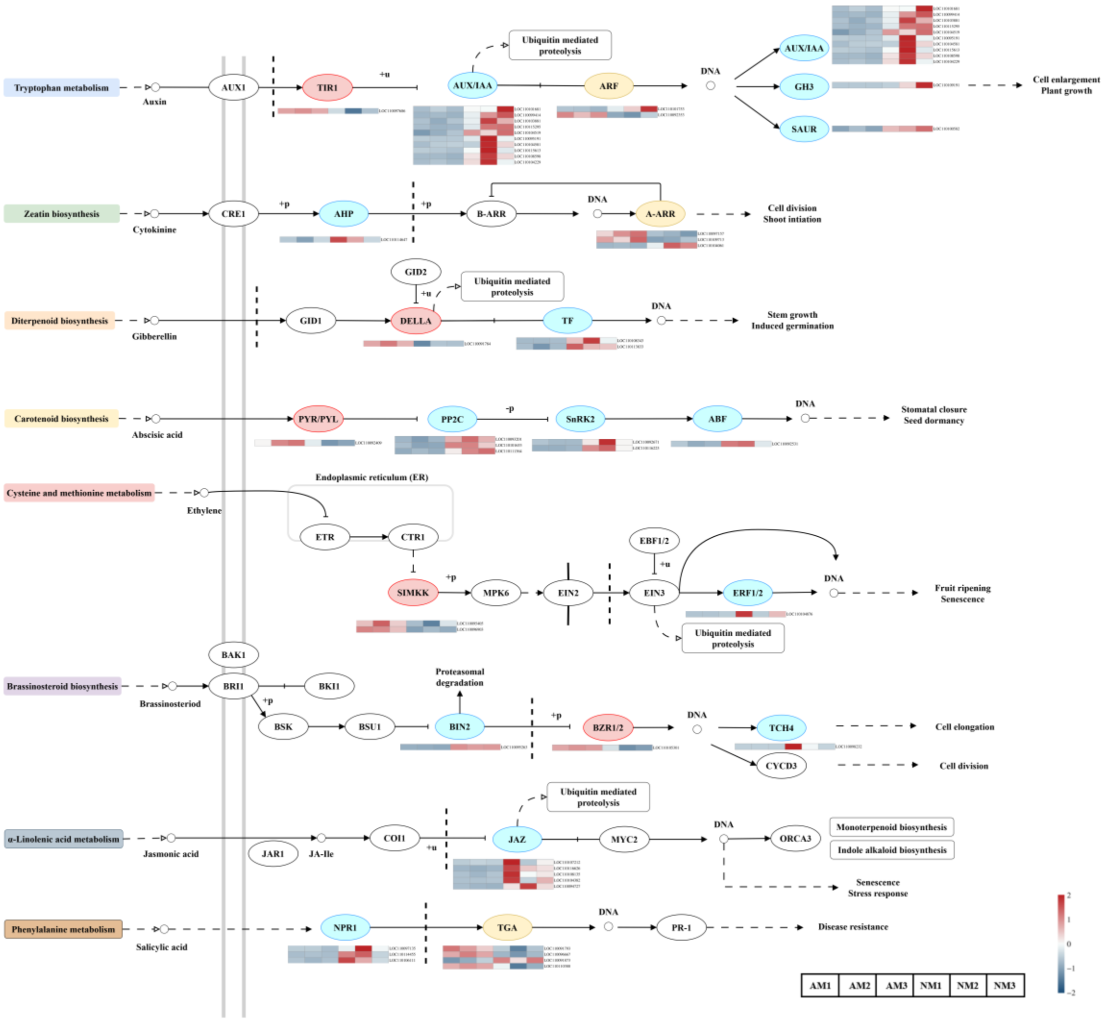

Supplementary Figure S4. The expression patterns of genes in the plant hormone signal transduction pathway of *D. officinale*.

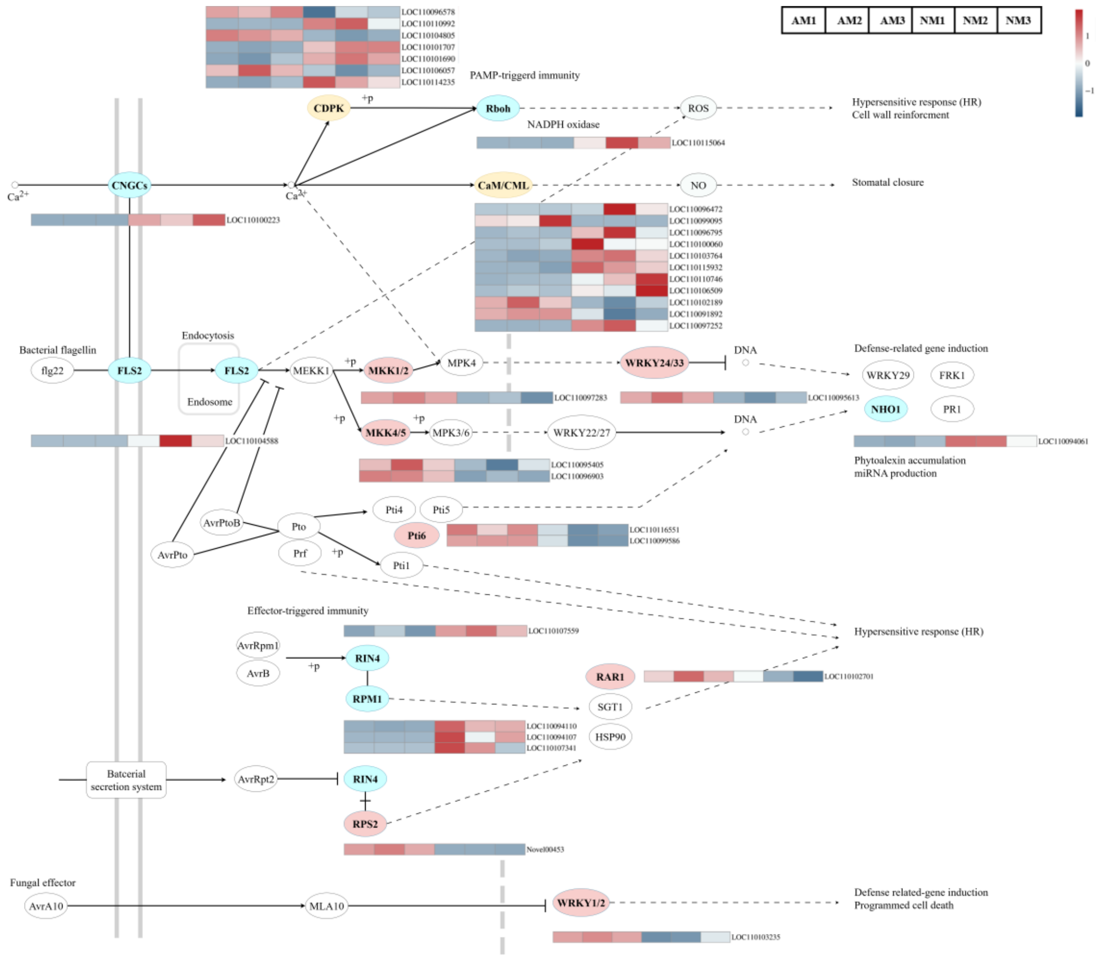

Supplementary Figure S5. The expression patterns of genes in the plant-pathogen interactions pathway of *D. officinale*.

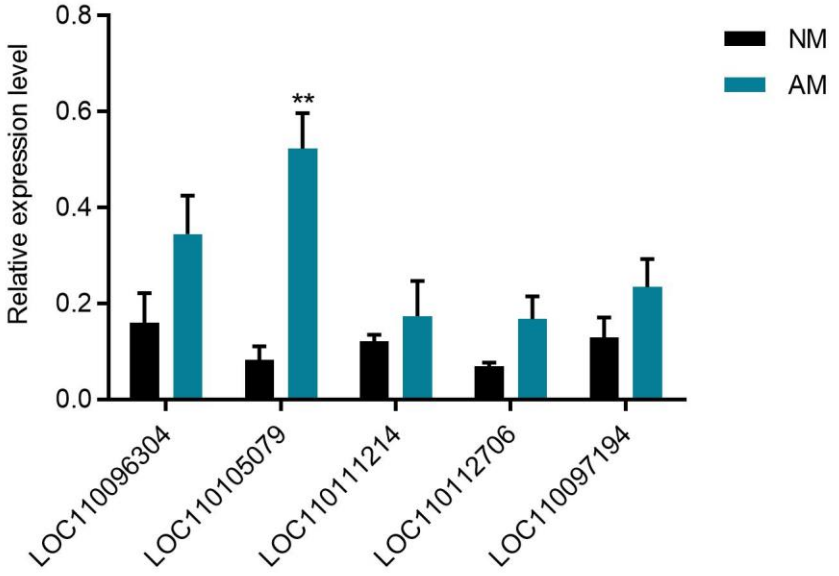

Supplementary Figure S6. This study analyzes the expression of DoUGT genes in response to arbuscular mycorrhizal (AM) symbiosis. Dendrobium officinale plants were inoculated with Rhizophagus irregularis (+AMF) or mock-inoculated (-AMF), after which samples were collected for quantitative reverse transcription polymerase chain reaction (qRT-PCR) analysis ($**P < 0.01$).

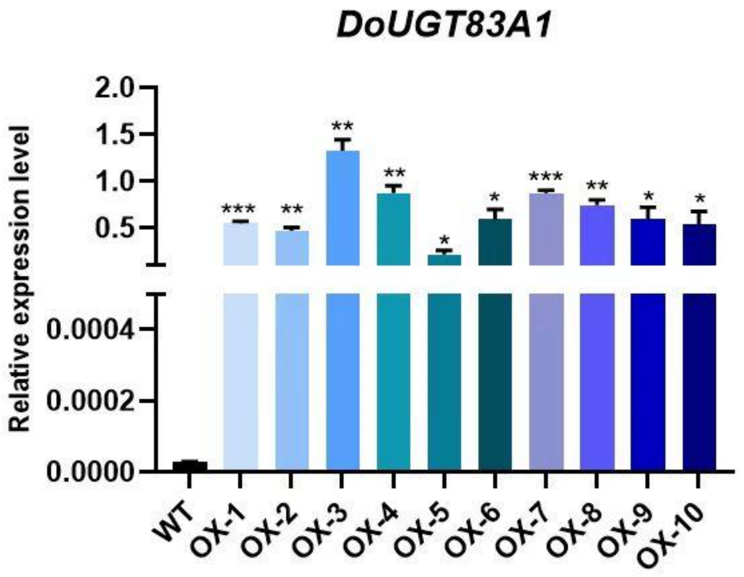

Supplementary Figure S7. DoUGT83A1 is overexpressed in the non-mycorrhizal roots of transgenic lines that overexpress DoUGT83A1. The designations OX1 to OX10 refer to the UGT83A1-OE1 through UGT83A1-OE10 lines, respectively. The values presented are the means of three biological replicates, accompanied by standard error (SE). Asterisks denote significant differences (*P < 0.05, **P < 0.01, ***P < 0.001).

Table S1. Summary of root transcriptome RNA-seq reads of *Dendrobium officinale*

Sample	Replicates	No.base in dataset (after QC)	No.clean reads	Uniquely mapped Ratio (%)	≥Q30(%)	GC Content(%)
AM	1	6,067,889,700	40,452,598	91.46	93.45	46.38
	2	6,321,205,800	42,141,372	91.06	93.24	46.63
	3	7,050,904,200	47,006,028	90.89	93.55	46.09
NM	1	6,424,395,600	42,829,304	92.01	93.11	45.88
	2	5,902,333,500	39,348,890	92.16	93.70	45.87
	3	6,631,716,000	44,211,440	92	93.24	45.78

AM : mycorrhizal symbiotic root system;NM :non-mycorrhizal symbiotic root system

Table S2. Number of differential expressed genes involved in each carbohydrate metabolic pathways.

Number	Pathway	DEGs Number	KO
1	Starch and sucrose metabolism	33	ko00500
2	Glycolysis/Gluconeogenesis	29	ko00010
3	Pentose phosphate pathway	20	ko00030
4	Pyruvate metabolism	19	ko00620
5	Fructose and mannose metabolism	16	ko00051
6	Glyoxylate and dicarboxylate metabolism	14	ko00630
7	Amino sugar and nucleotide sugar metabolism	14	ko00520
8	Inositol phosphate metabolism	12	ko00562
9	Pentose and glucuronate interconversions	12	ko00040
10	Ascorbate and aldarate metabolism	10	ko00053
11	Citrate cycle (TCA cycle)	8	ko00020
12	N-Glycan biosynthesis	8	ko00510
13	Galactose metabolism	7	ko00052
14	Glycosaminoglycan degradation	6	ko00531
15	Glycosylphosphatidylinositol (GPl)-anchor biosynthesis	4	ko00563
16	Other glycan degradation	3	ko00511
17	Other types of O-glycan biosynthesis	2	ko00514

Table S3. The key enzyme genes involved in polysaccharide synthesis in *D. officinale.*

EC	Enzyme	Abbreviation	Number
3.1.3.12	trehalose-phosphatase	TPP	6
3.1.3.11	fructose-1, 6-bisphosphatase	FBPase	4
3.1.3.46	fructose-2, 6-bisphosphatase		
3.2.1.21	beta-glucosidase	CB	4
3.2.1.4	endoglucanase	CB	3
2.4.1.15	trehalose phosphate synthase	TPS	3
2.7.1.11	6-phosphofructokinase	PFK	3
3.1.3.24	sucrose phosphatase	SPP	3
3.2.1.1	alpha-amylase	-	2
3.2.1.39	glucan endo-1,3-beta-D-glucosidase	BG	2
3.2.1.78	beta-mannanase	-	2
4.1.2.13	fructose-bisphosphate aldolase	FBA	2
2.4.1.13	sucrose synthase	SuSy	1
2.4.1.14	sucrose phosphate synthase	SPS	1
2.4.1.18	1,4-alpha-glucan branching enzyme	GBE	1
2.7.1.1	hexokinase	HXK	1
2.7.1.4	fructokinase	FRK	1
2.7.1.90	diphosphate--fructose-6-phosphate 1-phosphotransferase	PFP	1
2.7.7.13	UDP-glucose glycosyltransferase	UGGT	1
5.1.3.18	GDP-D-mannose 3', 5'-epimerase	GME	1
5.4.2.2	phosphoglucomutase	PGM	1

Table S4. Information on differentially expressed glycosyltransferases in *D. officinale.*

Gene ID	KEGG Annotation	Potential Interacting Transcription Factor
LOC110105079<br>*LOC110091880*	UDP-glycosyltransferase 83A1-like	HSF
LOC110096304	UDP-glycosyltransferase 88B1	
LOC110101924	UDP-glycosyltransferase 73C4-like	
LOC110101938	UDP-glycosyltransferase 73C4-like	
LOC110110985	UDP-glycosyltransferase 71K2-like	
LOC110111214	UDP-glycosyltransferase 73E1-like	
LOC110103890	probable glycosyltransferase At3g07620	ERF
LOC110112706	glycosyltransferase BC10-like	SBP
LOC110115451	probable xyloglucan glycosyltransferase 9	-
LOC110107105	probable xyloglucan glycosyltransferase 9	-
LOC110097194	glycosyltransferase family 92 protein Os08g0121900	

Table S5. List of the primers used for qRT-PCR analysis in this study.

**Gene**	**Forward Primer**	**Reverse Primer**
DoUGT83A1	ATGAGCTCCCCTCATGCACT	TTAAATTTCTCTTATAAAATC
qDoUGT83A1	CCAATTGTTTAACCAAAGCT	TTCAAAGCTTGAGATCTTGCC
DoActin	CCCTACCTCCTACCTCTGCG	GCAAACCCAGCCTTCACCAT
SlActin	TTCCGTTGCCCAGAGGTCCT	TCGCCCTTTGAAATCCACATC
SlPT4	GGTTTATCAGGGAATTTCTTGTTC	CAACAAATAC AATCATAGTA
qDoCSLA6	TCGTTCACAACCCCCAACTC	ACATGGAGGACCCAACCTCT
qDoPMM	GCTCCCAGAAAGGCTATAACTC	CTCCAACGACACCAACTGTAA
